# A positive spin: large language models can help directors evaluate programs through their patients' own words

**DOI:** 10.1097/PR9.0000000000001219

**Published:** 2024-12-24

**Authors:** Leah Russell Flaherty, Kendra H. Oliver

**Affiliations:** a Allegheny Health Network, Psychiatry and Behavioral Health Instiute, Pittsburgh, PA, USA; b Responsible Data Science, Office of the Provost, University of Pittsburgh, Pittsburgh, PA, USA; c ArtLab Studio LLC, Pittsburgh, PA, USA

**Keywords:** Pain psychology, Qualitative analysis, Thematic analysis, Generative artificial intelligence (GenAI), Large language models (LLM)

## Abstract

**Introduction:**

Interpretation and utilization of qualitative feedback from participants has immense value for program evaluation. Reliance on only quantitative data runs the risk of losing the lived patient experience, forcing their outcomes to fit into our predefined objectives.

**Objectives:**

Using large language models (LLMs), program directors may begin to employ rich, qualitative feedback expediently.

**Methods:**

This study provides an example of the feasibility of evaluating patient responses (n = 82) to Empowered Relief, a skill-based pain education class using LLMs. We utilized a dual-method analytical approach, with both LLM-assisted and supported manual thematic review.

**Results:**

The thematic analysis of qualitative data using ChatGPT yielded 7 major themes: (1) Use of Specific Audiofile; (2) Mindset; (3) Technique; (4) Community and Space; (5) Knowledge; (6) Tools and Approaches; and (7) Self-awareness.

**Conclusion:**

Findings from the LLM-derived analysis provided rich and unexpected information, valuable to the program and the field of pain psychology by employing the set of patients' own words to guide program evaluation. Program directors may benefit from evaluating treatment outcomes on a broader scale such as this rather than focusing solely on improvements in disability. These insights would only be uncovered with open-ended data, and although potentially more insights could emerge with the help of a qualitative research team, ChatGPT offered an ergonomic solution.

## Abstract 2

Supplemental Digital Content is Available in the Text.

The use of large language model thematic analysis of program feedback reveals benefits of a pain education program that quantitative data alone would not have elucidated.

## 1. Introduction

Qualitative feedback from patients has value for program evaluation.^2^ We select and use quantitative measures as a function of ease and standardization. However, reliance on only quantitative data risks missing the lived patient experience, forcing outcomes to fit into predefined objectives. Using large language models (LLMs), program directors may apply rich, qualitative feedback expediently.

Recently, the exploration of patients' own words led to an important linguistic change in the field of pain psychology. Analysis of negative patient responses to the term “pain catastrophizing” highlighted the importance of a shift away from the term.^24^ Evaluation of feedback provides justification for language or program alteration in a way that quantitative or anecdotal evidence may not. Ongoing advance of LLMs coupled with the increasing accessibility of computing power and data provide an opportunity for qualitative evaluation. With responsible use, LLMs, specifically ChatGPT, can save time and money, mitigate bias, expand the empirical base, and provide patient data–based rationale for program modifications.

This article provides an example of how program directors employed an LLM as a collaborative tool for thematic analysis to evaluate feedback. We document the approach that was used and highlight some of the limitations and considerations for the responsible use of LLMs for program administrators to leverage qualitative feedback. When used responsibly, LLMs can benefit program directors who may not have resources to evaluate qualitative data.

## 2. Methods

Through Allegheny Health Network, 82 individuals enrolled in Empowered Relief and completed a postclass satisfaction survey. Empowered Relief is a 2-hour, evidenced-based, single-session pain intervention created at the Stanford University.^12^ One-month post class, participants completed a 16-item satisfaction survey. The one open-ended question used in this analysis was, “What was most helpful, or did you learn anything in particular that might be helpful to you in the future?”

To evaluate patient responses, a dual-method analytical approach used both LLM-assisted and supported manual thematic review. The foundational approach is based on inductive thematic analysis, a method widely used in qualitative research for identifying, analyzing, and reporting patterns within data.^7^ Qualitative inquiry can improve the description and explanation of complex, real-world phenomena pertinent to health services research.^6,7,8^

Experiments with ChatGPT have noted its ability to facilitate faster analysis of qualitative data sets and adaptability to understand and generate sociological knowledge, organize codes into clusters, and assist in identifying patterns or connections.^19^ All responses were uploaded to ChatGPT to generate codes in a deidentified format. The prompt, shown in Table 1, was engineered based on existing examples and resources available for prompt engineering.^21,25,26^ To conduct the LLM-assisted thematic analysis, R1 (Oliver) accessed OpenAI's ChatGPT interface and established prompt structure using the paid subscription of GPT-4. *There were no inclusion/exclusion criteria. All data available based on open text response to a single question was used.* These codes were evaluated manually before being manually organized into themes, assisted by the LLM by R1 with the idea to minimize the number of themes without distorting concepts. Results were reviewed by both R1 and R2 (Flaherty). Proprietary information and any potentially identifying details were removed.

**Table 1:** ChatGPT thematic analysis.

Prompt: “You are a qualitative researcher starting to do a thematic analysis. I have uploaded responses from participants in a program. You will do quantitative, initial, or open coding all the same. The text is individual answers to the question, “What was most helpful, or did you learn anything in particular that might be helpful to you in the future?” I want the code to be detailed and descriptive. I want you to apply codes to sentences or parts of sentences from the document attached and develop a list of codes. Please provide at least 5 sample quotes related to each code you identify from the document.” (modified from …) Full article: Can ChatGPT Evaluate Plans? (tandfonline.com) Hacking by the prompt: Innovative ways to utilize ChatGPT for evaluators - Ferretti - 2023 - New Directions for Evaluation - Wiley Online Library Follow-up Prompt: After being prompted with, “Using the same format as your previous responses, generate more codes with at least 5 examples from the document above,” 15 codes were developed. This was repeated at least twice to get a minimum of 15 codes total. These codes were evaluated manually before being manually organized into themes. The original Chat conversation can be found below. Full Exchange: https://chat.openai.com/share/4696cbb4-2d98-46b6-96bd-d85f5ee2933c		
1	Use of audio tools for pain management	“The audio [] was helpful.” “Most helpful was the idea of using binaural sound meditation.” “I also prefer the audio, especially at night.” “The calm, steady voice of the narrator was effective.” “The binaural relaxation was my favorite takeaway.”
2. Mindset	Empowerment and control over pain	“I got a feeling of empowerment, which I feel that I have been able to keep with me.” “Using my own abilities to control pain.” “That I can partially control my pain, especially when I am at rest.” “Offered additional tools I did not have and gave me a better understanding that I was not alone in dealing with pain.” “For me, it came at a perfect time and has been very helpful.”
	Positive thinking and focus shift	“Focus on trying to enjoy things a little better and not focus on pain.” “Optimistic and hopeful message. Reminder to use positive self-talk.” “Redirect thinking about pain and its consequences.” “Making me focus and think about things that help reduce my pain.” ***“I learned a lot - but the best has been my use of the meditation app.***”
3. Technique	Relaxation techniques	“I think it was helpful to just learn the relaxation techniques.” “The relaxation exercises.” “The relaxation tape and positive thinking.” “The instructor was pleasant and helpful. The relaxation techniques will help in the future.” “Helps me to relax and lessen the pain when trying to get to sleep.”
	Meditation and mindfulness techniques	“Considering meditation like medicine helped me immensely.” “I guess, the meditation, and breathing techniques.” “Mental outlook and the meditation.” “Understanding the research, and finding appropriate meditations to help calm my nervous system.” ***“The entire process is new to me. I had not tried anything to reduce the pain other than the occasional use of Tylenol.***”
	Breathing techniques	“I thought the breathing exercises were of a big help to me.” “The power of my breath and how I can distract my brain while experiencing pain.” “Just taking time to breathe really helps me and focusing on it.” “I remember to use my breathing to relax during stressful times.” “Focusing on slowing my breath. Many years ago, I realized that a key indicator of my pain ramping up was shallow breathing.”
4. Community and space	Community and shared experience	“Being able to share what we're going through.” “I realized that there were a lot of other people who had the same issues.” “Knowing that others also feel the same way I do, and reframing the shame I feel when I can't do things all the time.” “It was helpful learning that pain not only affects me emotionally but physically. It helped me to be in a group to see that others are dealing with pain like me; it made me feel less isolated.” ***“The class motivated me to actively continue to search for other ways to help myself.***”
	Emotional coping and management	“Offered additional tools I did not have and gave me a better understanding that I was not alone in dealing with pain.” “Knowing that others also feel the same way I do and reframing the shame I feel when I can't do things all the time.” “That your emotions worsen pain.” “The relaxation tape and positive thinking.” “I am under extreme stress at this point in my life, so spending time with the binaural recording and learning the other coping ideas have been good things to take away from the program.”
5. Knowledge	Understanding pain and its effects	“Understanding the power of the brain. I never thought about it before this session.” “You mean the class, right. It was informative and helpful to understand the effects chronic pain has on your brain and life.” “The science of pain and its effect on the brain.” “The science behind the physical changes with anxiety and how similar they are to the pain response.” “I learned that your brain can not process pain and other thoughts at the same time, so if you can get your brain to focus on something else it will help with pain relief.”
	Educational impact and knowledge gain	“Understanding the research, and finding appropriate meditations to help calm my nervous system.” “It was informative and helpful to understand the effects chronic pain has on your brain and life.” “The science of pain, and its effect on the brain.” “The science behind the physical changes with anxiety and how similar they are to the pain response.” “Learning how to rethink pain.”
6. Tools and approaches	Coping strategies and practical tools	“The tips to help lower stress and cope with pain.” “The worksheets helping us to lay out an action plan, reframing unhelpful pain thoughts, reframing to 'best friend talk,' and coming up with a list of self-soothing actions.” “A concrete plan - something constructive to do.” “Learning how to rethink pain.” “The after class materials were very helpful to get through pain afterwards.”
	Personal growth and self-reflection	“Making me focus and think about things that help reduce my pain.” “I got a feeling of empowerment which I feel that I have been able to keep with me.” “The worksheets helping us to lay out an action plan, reframing unhelpful pain thoughts, reframing to 'best friend talk,' and coming up with a list of self-soothing actions.” ***“The segment dealing with the binaural audio file. Any additional info regarding this type of therapy would be helpful.”*** “The study on the use of binaural beats. Also filling out the worksheet before the workshop caused me to acknowledge how much guilt I felt about not being able to be the partner I want to be to my husband because of my pain and how it changed our lives.”
	Technological tools for pain management	“The audio App was helpful.” “Most helpful was the idea of using binaural sound meditation.” “The binaural relaxation was my favorite takeaway.” “The binaural meditation app that was provided postmeeting has been most helpful to me. I use it daily.” “The audio file is a great way to concentrate and be mindful about relaxing.”
7. Self-awareness	Self-assessment and body awareness	“Importance of self-assessment and noting how my body responds to different activities.” “I learned to recognize my pain and actually respond to it.” “I learned to listen to my body and recognize symptoms of anxiety as pain.” “Taking the time to refocus.” “I got a feeling of empowerment which I feel that I have been able to keep with me.”
	Adapting to physical limitations and recovery	“For me, it came at a perfect time and has been very helpful. It is hard to find time to squeeze everything into your day, with PT 2–3 times a day, although when I do I feel so much better.” ***“I had robot-assisted surgery and the pain in a lot less than I expected, I would say it has never passed the 4 level and that it is in the 2–3 level.***” “Turns out I had Lyme disease and was treated, which reduced a lot. I also had low cortisol, secondary adrenal insufficiency and am now on medication, which also reduced some pain.” “The entire process is new to me. I had not tried anything to reduce the pain other than the occasional use of Tylenol.” ***“I wish I could listen to the calming tones, but I cannot tolerate them with my migraines. Maybe in the future, I could try again.***”

## 3. Results

The thematic analysis of qualitative data using chat GPT yielded 7 major themes: (1) Use of Specific Audiofile; (2) Mindset; (3) Technique; (4) Community and Space; (5) Knowledge; (6) Tools and Approaches; and (7) Self-awareness. R1 and R2 reviewed the output developed by the algorithm to ensure that none were hallucinations and fit the code and theme.^22^

## 4. Discussion

Findings from the LLM-derived analysis provided rich and unexpected information, valuable to the program and the field of pain psychology by employing patients' own words.

Within the theme of *Mindset,* participants reported the importance of a newfound sense of control, “Using my own abilities to control pain.” By learning cognitive behavioral therapy skills to redirect thoughts, participants felt more hopeful and powerful. Increasing confidence and efficacy in one's ability to control their experience may be protective against pain intensity and comorbid conditions that often accompany chronic pain.^15,18^

Within *Techniques and Knowledge*, participants reported a newfound ability to regulate systemic arousal and a shift in thinking of this as worthwhile, “considering meditation like medicine helped me immensely.” The importance of health literacy is highlighted in this theme, as patients learn more about the pathophysiology of chronic pain, they become more able to cope with and manage their condition. This benefit cannot be overstated as low health literacy has been linked in the literature with opioid misuse, pain severity, and pain disability among adults with chronic pain.^20^

For *Community and Space,* participants felt merit in being in a cooperative space and reported a sense of motivation in a shared goal toward pain-relief, “knowing that others feel the same way I do refram[ed] the shame I feel.” Patients with chronic pain often report struggling with social isolation which has been found to negatively impact both physical and emotional treatment outcomes.^4,16^ This theme is particularly appealing when considering 85% of participants who completed the survey attended ER on-line.

In *Self-Awareness,* participants reported the ability to listen to their bodies and mindfully take time to shift focus, “Importance of self-assessment and noting how my body responds to different activities.” As people with chronic pain learn to attend to the distinction of what is and what is not painful, they may experience improvements in kinesophobia. This has been linked in the literature with positive implications in both physical and mental health of those coping with chronic pain.^9^

Empowered Relief has been found to decrease pain catastrophizing, pain intensity, depression, anxiety, pain interference, sleep disturbance, and pain bothersomeness.^12,13^ By using ChatGPT to evaluate patient responses, a more linguistically positive and richer interpretation of patient perception emerged (power, ability, control, focus, hopeful, action, and understanding) without predefined metrics. Evaluating treatment outcomes on a broader scale through open-response text rather than solely focusing on improvements in disability may be beneficial.^3^ Although potentially more insights could emerge with the help of a qualitative research team, ChatGPT offered an ergonomic solution to this end.

An unanticipated finding was the absence of reference to stigma associated with mental health care for chronic pain. The literature indicates a stigma regarding psychological interventions for pain held by both patient and providers.^14^ Despite the evidence for effectiveness, psychological treatments for chronic pain are often a last resort.^11^ Perhaps, stigmatizing views about these treatments are held more by providers than by patients themselves.

There are many things to consider when opting for this approach to evaluate text data. It has been noted that LLMs tend to generate more descriptive rather than interpretive responses, resulting in less inferential reasoning as compared with human analysts who apply interpretation to data.^19^ This reflects a broader issue in AI-driven analysis, often called the “black box” problem, whereby the processes by which the model arrives at its conclusions are not fully transparent. One might argue that this issue mirrors the cognitive process in human analysis, which can similarly be considered a “black box” because of the subjective and sometimes inexplicable nature of human cognition and interpretation. Since feedback from surveys would typically be read by a trained psychologist, this approach provided an opportunity to review the data through a novel “black-box.” However, many are exploring and finding utility for ChatGPT in qualitative data analysis, including medical practice evaluation, for code generation, and clarifying prompt generation.^17,23,26^

If program administrators use LLMs to elucidate novel insights, the program director should be familiar with the considerations for the appropriate, rigorous, and reliable use of AI in research practice.^10^ The skills used in this type of analysis include prompt engineering, a field that is rapidly evolving. In addition, it is essential to recognize that sending prompts and data to ChatGPT or other cloud-based large language models, like ChatGPT, could potentially divulge information on patients and respondents that then becomes part of future releases and responses. Although many in the AI field are actively defining responsible data science, intention should be on communication and upskills.^5^

Validation of results is also key to prevent misinterpretation of LLM coding, supporting measurement validity, and identifying the presence of LLM hallucinations.^1^ In this case study, while manually validating the results, reviewers found 4 example quotes that did not necessarily match the theme (bold and italicized in Table 1) but none were hallucinations. Beyond these limitations, ChatGPT, a new and evolving tool, was able to mirror participant's own words, and offered a strengths-based evaluation of the program. LLMs provide an opportunity to efficiently integrate qualitative insights offered from direct reading with greater ease and speed of computational approaches, providing novel insights for program directors if approached responsibly. The use of the LLMs for initial coding in our example of the thematic analysis of program feedback reveals the benefits and considerations for AI-driven tools in synthesizing qualitative data. Quantitative data alone would not have elucidated increases in power, cognitive, and somatic awareness, as well as the sense of community fostered by participants in a pain education program (ChatGPT_Themes—Supplementary Material, http://links.lww.com/PR9/A270).

## Disclosures

The authors declare that they have no conflict of interest.

## Appendix A. Supplemental digital content

Supplemental digital content associated with this article can be found online at http://links.lww.com/PR9/A270.

## Supplementary Material

SUPPLEMENTARY MATERIAL
