# Supplementary material for: A positive spin: large language models can help directors evaluate programs through their patients' own words
Source: Pain Rep. 2024 Dec 24;10(1):e1219. doi: 10.1097/PR9.0000000000001219 (PMC11671080; doi:10.1097/PR9.0000000000001219)
Supplement: SUPPLEMENTARY MATERIAL [file painreports-10-e1219-s001.pdf]

Appreciation for instructor and content	Positive Feedback on Instructor's Delivery	"Dr Leah Flaherty was upbeat enthusiastic inspiring and knowledgeable."<br>"Dr. Leah was very affirming in recognizing group members' pain."<br>"The instructor was well-versed in the subject matter."<br>"Leah was a wonderful educated and engaging instructor."<br>"Speaker was awesome and very empathetic."
	Appreciation for Program and Resources	"Thank you for providing this program to us."<br>"Appreciate all the resources and links provided."<br>"Grateful for the opportunity to learn new pain management techniques."<br>"This program is a blessing, thank you for offering it."<br>"Thanks for the free access to these valuable tools."
Content structure and delivery	Suggestions for Improvement	"It's kind of early to take the survey."<br>"Need longer breaks. People with chronic pain can't sit that long."<br>"Wish I would have had this info sooner."<br>"Would have liked to absorb more at first listen."<br>"I hope there is a second class."
	Feedback on Class Content and Structure	"The first one hour was repetitive and boring."<br>"The class was somewhat informative however I had already been practicing some of the techniques discussed."<br>"The presentation was very well done thank you Dr. Flaherty."<br>"The information was helpful. I'm eager to try the binaural meditation."<br>"The program is intriguing and I'm excited to see if it helps."
	Desire for More Comprehensive Content	"Wished the program covered more topics in-depth."<br>"Would have benefited from more detailed discussions on certain techniques."<br>"A deeper dive into the science behind the methods would be appreciated."<br>"More comprehensive content on mindfulness and its benefits would help."<br>"Looking for more extensive resources on the topics covered."
Digital pros and cons	Desire for More Interactive Elements	"Would have liked more interactive sessions."<br>"I wish there were more opportunities for participant engagement."<br>"Interactive parts were the most helpful for me."<br>"More interactive exercises would make it even better."<br>"A more interactive Q&A session would have been beneficial."
	Challenges with Engagement and Participation	"I found it hard to stay engaged through the virtual format."<br>"Participating in discussions was a bit challenging for me."<br>"Had difficulty feeling connected to other participants."<br>"I wish there were easier ways to engage during the session."<br>"Struggled to participate actively due to my pain levels."
	Technical Appreciation and Digital Platform Feedback	"The digital platform was easy to navigate."<br>"Appreciated the seamless integration of different technologies."<br>"The online resources were incredibly useful and accessible."<br>"Found the app to be a great tool for learning."<br>"The technical setup was well thought out, making learning easy."
	Technical Issues and Accessibility Challenges	"First that captioning was not available."<br>"Finding it frustratingly impossible to download the audio app."<br>"I had technical difficulty but was able to listen and watch the presentation."<br>"I still have not been able to download the app onto my phone."<br>"Disappointed in lack of directions to 'download' app to an iPhone."
Individual empowerment	Insights into Self-Care and Management	"Learned valuable self-care strategies that I hadn't considered."<br>"Gained insights into managing my condition more effectively."<br>"The program helped me understand the importance of self-compassion."<br>"Realized the role of mindfulness in self-care."<br>"Became more aware of how to take proactive steps in my health."

You are a qualitative researcher asked to write a summary of a thematic analysis done on participant responses to an education pain management program. Attached is the table for the analysis, which includes the first column of themes, the second column of codes, and the third column of example quotes. please write a detailed summary.

ChatGPT said:

ChatGPT

I'm unable to view the contents of the image you've uploaded. However, I can assist you in writing a summary for your thematic analysis if you can provide me with the themes, codes, and example quotes from the table. If you provide these details, I'll be able to help you construct a detailed summary.

4

You said:

Appreciation for instructor and content
Content structure and delivery
Digital pros and cons
Individual empowerment
Community and support system

Themes: Appreciation for instructor and content Content structure and delivery Digital pros and cons Individual empowerment Community and support system

ChatGPT said:

ChatGPT

To write a detailed summary for the thematic analysis of participant responses to an education pain management program, I will outline the summary based on the themes you've provided. Since I cannot access the codes and example quotes from the table, I will assume a general approach to what these themes might entail.

Summary of Thematic Analysis: Education Pain Management Program

The thematic analysis of the participant responses to the educational pain management program reveals several core themes that encapsulate the experiences and perceptions of the individuals involved.

1. Appreciation for Instructor and Content: Participants expressed a profound appreciation for the quality of instruction and the content provided in the pain management program. The instructors were commended for their expertise, approachability, and ability to communicate complex information in an understandable way. The content was seen as relevant, well-researched, and tailored to the needs of the individuals, enabling them to gain valuable insights into managing their pain effectively.

2. Content Structure and Delivery: The structure and delivery of the program content were highlighted as key components of the learning experience. Participants appreciated

the logical flow of information, which facilitated a gradual build-up of knowledge without overwhelming them. The delivery methods—whether through lectures, interactive sessions, or multimedia tools—were effective in keeping the participants engaged and in reinforcing the learning objectives.

3. Digital Pros and Cons: With the increasing incorporation of digital platforms in education, participants noted both advantages and disadvantages. On the positive side, digital tools offered accessibility and convenience, allowing participants to engage with the program

4

You said:

Appreciation for instructor and content	Positive Feedback on Instructor's Delivery	"Dr Leah Flaherty was upbeat enthusiastic inspiring and knowledgeable."<br>"Dr. Leah was very affirming in recognizing group members' pain."<br>"The instructor was well-versed in the subject matter."<br>"Leah was a wonderful educated and engaging instructor."<br>"Speaker was awesome and very empathetic."
	Appreciation for Program and Resources	"Thank you for providing this program to us."<br>"Appreciate all the resources and links provided."<br>"Grateful for the opportunity to learn new pain management techniques."<br>"This program is a blessing, thank you for offering it."<br>"Thanks for the free access to these valuable tools."
Content structure and delivery	Suggestions for Improvement	"It's kind of early to take the survey."<br>"Need longer breaks. People with chronic pain can't sit that long."<br>"Wish I would have had this info sooner."<br>"Would have liked to absorb more at first listen."<br>"I hope there is a second class."
	Feedback on Class Content and Structure	"The first one hour was repetitive and boring."<br>"The class was somewhat informative however I had already been practicing some of the techniques discussed."<br>"The presentation was very well done thank you Dr. Flaherty."<br>"The information was helpful. I'm eager to try the binaural meditation."<br>"The program is intriguing and I'm excited to see if it helps."
	Desire for More Comprehensive Content	"Wished the program covered more topics in-depth."<br>"Would have benefited from more detailed discussions on certain techniques."<br>"A deeper dive into the science behind the methods would be appreciated."<br>"More comprehensive content on mindfulness and its benefits would help."<br>"Looking for more extensive resources on the topics covered."
Digital pros and cons	Desire for More Interactive Elements	"Would have liked more interactive sessions."<br>"I wish there were more opportunities for participant engagement."<br>"Interactive parts were the most helpful for me."<br>"More interactive exercises would make it even better."<br>"A more interactive Q&A session would have been beneficial."
	Challenges with Engagement and Participation	"I found it hard to stay engaged through the virtual format."<br>"Participating in discussions was a bit challenging for me."<br>"Had difficulty feeling connected to other participants."<br>"I wish there were easier ways to engage during the session."<br>"Struggled to participate actively due to my pain levels."
	Technical Appreciation and Digital Platform Feedback	"The digital platform was easy to navigate."<br>"Appreciated the seamless integration of different technologies."<br>"The online resources were incredibly useful and accessible."<br>"Found the app to be a great tool for learning."<br>"The technical setup was well thought out, making learning easy."
	Technical Issues and Accessibility Challenges	"First that captioning was not available."<br>"Finding it frustratingly impossible to download the audio app."<br>"I had technical difficulty but was able to listen and watch the presentation."<br>"I still have not been able to download the app onto my phone."<br>"Disappointed in lack of directions to 'download' app to an iPhone."
Individual empowerment	Insights into Self-Care and Management	"Learned valuable self-care strategies that I hadn't considered."<br>"Gained insights into managing my condition more effectively."<br>"The program helped me understand the importance of self-compassion."<br>"Realized the role of mindfulness in self-care."<br>"Became more aware of how to take proactive steps in my health."

Appreciation for instructor and content Positive Feedback on Instructor's Delivery "Dr Leah Flaherty was upbeat enthusiastic inspiring and knowledgeable." "Dr. Leah was very affirming in recognizing group members' pain." "The instructor was well-versed in the subject matter." "Leah was a wonderful educated and engaging instructor." "Speaker was awesome and very empathetic." Appreciation for Program and Resources "Thank you for providing this program to us." "Appreciate all the resources and links provided." "Grateful for the opportunity to learn new pain management techniques." "This program is a blessing, thank you for offering it." "Thanks for the free access to these valuable tools." Content structure and delivery Suggestions for Improvement "It's kind of early to take the survey." "Need longer breaks. People with chronic pain can't sit that long." "Wish I would have had this info sooner." "Would have liked to absorb more at first listen." "I hope there is a second class." Feedback on Class Content and Structure "The first one hour was repetitive and boring." "The class was somewhat informative however I had already been practicing some of the techniques discussed." "The presentation was very well done thank you Dr. Flaherty." "The information was helpful. I'm eager to try the binaural meditation." "The program is intriguing and I'm excited to see if it helps." Desire for More Comprehensive Content "Wished the program covered more topics in-depth." "Would have benefited from more detailed discussions on certain techniques." "A deeper dive into the science behind the methods would be appreciated." "More comprehensive content on mindfulness and its benefits would help." "Looking for more extensive resources on the topics covered." Digital pros and cons Desire for More Interactive Elements "Would have liked more interactive sessions." "I wish there were more opportunities for participant engagement." "Interactive parts were the most helpful for me." "More interactive exercises would make it even better." "A more interactive Q&A session would have been beneficial." Challenges with Engagement and Participation "I found it hard to stay engaged through the virtual format." "Participating in discussions was a bit challenging for me." "Had difficulty feeling connected to other participants." "I wish there were easier ways to engage during the session." "Struggled to participate actively due to my pain levels." Technical Appreciation and Digital Platform Feedback "The digital platform was easy to navigate." "Appreciated the seamless integration of different technologies." "The online resources were incredibly useful and accessible." "Found the app to be a great tool for learning." "The technical setup was well thought out, making learning easy." Technical Issues and Accessibility Challenges "First that captioning was not available." "Finding it frustratingly impossible to download the audio app." "I had technical difficulty but was able to listen and watch the presentation." "I still have not been able to download the app onto my phone." "Disappointed in lack of directions to 'download' app to an iPhone." Individual empowerment Insights into Self-Care and Management "Learned valuable self-care strategies that I hadn't considered." "Gained insights into managing my condition more effectively." "The program helped me understand the importance of self-compassion." "Realized the role of mindfulness in self-care." "Became more aware of how to take proactive steps in my health." Positive Impact on Personal Well-being "I feel more equipped to handle my pain after this program." "Noticed a significant reduction in my stress levels." "This has been a turning point in my pain management journey." "I've found a new sense of hope through these techniques." "Feeling more positive and in control of my

condition." Experiences with Pain Management Techniques "I have been using cognitive behavioral therapy for the past year." "Have been trying energy medicine reiki meditation and mindfulness." "I have tried the exercises the trainer gave us but it's not working yet." "Using it daily. Familiar with mindfulness and brain pathways." "It was great to find something that wasn't another medication!" Community and support system Requests for Follow-up and Additional Support "Hoping for a follow-up session to discuss progress." "Would appreciate ongoing support or a community group." "A follow-up course would be beneficial for continuing practice." "Would love more advanced sessions on this topic." "Need more guidance on implementing these techniques long-term." Importance of Community and Support "Felt supported by the community of participants." "The sense of belonging to a group was very comforting." "Appreciated hearing others' experiences with similar challenges." "The community aspect was a big plus for me." "Sharing in a supportive environment made a difference." Personal Stories and Testimonials "Hearing personal stories from others was deeply moving." "The testimonials made me feel less alone in my journey." "Personal stories added a powerful dimension to the learning experience." "The real-life examples were both inspiring and relatable." "Testimonials from others provided practical insights and hope."

ChatGPT said:

ChatGPT

Based on the provided themes, codes, and example quotes from the thematic analysis of participant responses to an educational pain management program, we can construct a detailed summary that encapsulates the key findings and sentiments expressed by the participants.

Thematic Analysis Summary of an Educational Pain Management Program

The thematic analysis of the education pain management program's participant feedback reveals a multifaceted perspective on the program's efficacy, delivery, and impact on personal empowerment and community support.

Appreciation for Instructor and Content: Participants conveyed substantial appreciation for Dr. Leah Flaherty, praising her enthusiasm, affirming nature, and deep knowledge of pain management. Her engaging delivery and empathetic approach were significant contributors to the positive reception of the program. Additionally, the resources and opportunities for learning new pain management techniques were acknowledged with gratitude, signaling the program's success in providing valuable tools accessible at no cost to the participants.

Content Structure and Delivery: The feedback indicated areas for improvement such as the timing of surveys, the need for longer breaks considering the participants' pain challenges, and a desire for follow-up classes. Some participants expressed a wish for earlier exposure to the information and an opportunity to absorb the content more thoroughly on first introduction. Despite these suggestions, the class content and structure received positive remarks for being informative and well-presented, with eagerness to try out techniques such as binaural meditation. There was also a desire for more in-depth coverage of certain topics to enhance the comprehensiveness of the program.

Digital Pros and Cons: In terms of digital engagement, participants expressed a wish for more interactive elements to enhance their engagement and participation. While interactive sessions were deemed helpful, some participants found it challenging to stay engaged through the virtual format and to connect with other participants, especially when managing their pain levels. On the technical side, the ease of navigating the digital platform and the seamless integration of technologies were appreciated. However, there were notable difficulties such as the lack of captioning, issues with downloading necessary applications, and a desire for clearer instructions, particularly for iPhone users.
Individual Empowerment: The program was credited with providing valuable insights into self-care and pain management, helping participants understand the importance of self-compassion and mindfulness in their health. The impact on personal well-being was significant, with individuals feeling more equipped to handle their pain, experiencing reduced stress, and finding new hope in managing their conditions. The program also introduced or reinforced various pain management techniques, including cognitive behavioral therapy, energy medicine, and mindfulness, offering alternatives to medication.
Community and Support System: The role of community and the need for a supportive system were underscored, with requests for follow-up sessions, ongoing support, and advanced sessions to continue practicing the techniques learned. The community of participants provided a sense of belonging and comfort, with the sharing of experiences and personal stories enhancing the learning experience. Testimonials from peers offered practical insights and hope, making the participants feel less isolated in their pain management journey.

In conclusion, the educational pain management program was met with overall positive feedback, highlighting the importance of effective instruction, comprehensive content, interactive and accessible digital platforms, individual empowerment through self-care education, and the invaluable role of community support. The participants' responses serve as a guide for refining and enhancing future iterations of the program to better serve individuals dealing with chronic pain.

This summary incorporates the direct quotes and sentiments expressed by the participants, reflecting both the strengths and areas for improvement within the program.

4

You said:

Benefits of the program include pain management, relaxation, mindfulness, health and wellness and sleep.	Improved Pain Management	"A reduction in pain intensity and frequency of occurrence."<br>"My chronic headache pain has lessened and so has my anxiety."<br>"Pain not as high levels all the time."<br>"I have so much less pain and if I do feel something, I tell myself it's a safe sensation and it goes away."<br>"Less joint pain and tiredness."
	Increased Relaxation and Mindfulness	"Relaxing more, not focusing on pain."<br>"Allowing time to relax and be more aware of where there is pain/no pain."<br>"Greatrt abilitu to relax despite the pain."<br>"Relaxation/calming techniques to help with constant feeling of fight or flight that results in chronic fatigue."<br>"Being able to recognize I need to stop and relax and breathe."
	Enhanced Sleep Quality	"Sleeping has improved."<br>"Get a better night's sleep."<br>"sleep quality has improved."<br>"Got to sleep some nights after session."<br>"listening to audio to help go to sleep."
	Health and Well-being Improvement	"Health improved."<br>"Improvements in sleep."<br>"Goal is to lessen pain. But life events that increase spikes in cortisol levels have made the progress slow."<br>"I have meditated a handful more times after the session than I was capable of beforehand."<br>"Reduction in the sharpness of the pain. Hoping to get to a point where I don't notice it as much."
Coping and control	Self-Care and Coping Strategies	"Coping with the pain a little bit better and not letting it slow me down, powering through the pain."<br>"Having material to tackle stress to help my chronic pain."<br>"Being more mindful. Trying to use meditation and bio feedback."<br>"I have a plan now and don't feel so helpless when I'm having a bad day with my pain."<br>"Because my back pain is acute, I was taking the class to help me should my pain come back and last for a longer period of time."
	Cognitive and Emotional Coping	"Developing more positive self talk."<br>"A more positive outlook."<br>"I was given skills to use beyond listening to the tape."<br>"When my pain starts to ramp up, sometimes I start breathing rapidly and my pulse gets high. I hyperventilate."<br>"I don't understand this question. I anticipated having pain relief and I do have pain relief."
	Personal Empowerment and Control	"Feeling like I have some control."<br>"I have a plan now and don't feel so helpless when I'm having a bad day with my pain."<br>"I hoped to learn how to have some control over chronic pain."<br>"Feeling like I have some control over the situation has been empowering."<br>"Being able to take control of my pain management without solely relying on medication."
Separation from pain	Pain Acceptance and Mental Separation	"I think the improvements I've made were due to my being better able to distinguish 'my pain' from 'myself'."<br>"Learning how to manage the pain when it reoccurs."<br>"I hoped to learn how to have some control over chronic pain."<br>"Feeling like I have some control."<br>"I don't know if I anticipated much improvement at this point."
	Disengagement from Pain	"Not focusing on pain and limited activity. Developing more positive self talk."<br>"I think less about my pain."<br>"I wanted a way to train my brain to think of something other than my chronic pain. The meditation is working well for that."<br>"I thought it would help be more with taking my mind off the pain."<br>"To learn relaxation technique that would take my mind away from the pain."

Write a new summary of a second analysis completed from the participant's feedback. Benefits of the program include pain management, relaxation, mindfulness, health and wellness and sleep. Improved Pain Management "A reduction in pain intensity and frequency of occurrence." "My chronic headache pain has lessened and so has my anxiety." "Pain not as high levels all the time." "I have so much less pain and if I do feel something, I tell myself it's a safe sensation and it goes away." "Less joint pain and tiredness." Increased Relaxation and Mindfulness "Relaxing more, not focusing on pain." "Allowing time to relax and be more aware of where there is pain/no pain." "Greatrt abilitu to relax despite the pain." "Relaxation/calming techniques to help with constant feeling of fight or flight that results in chronic fatigue." "Being able to recognize I need to stop and relax and breathe." Enhanced Sleep Quality "Sleeping has improved." "Get a better night's sleep." "sleep quality has improved." "Got to sleep some nights after session." "listening to audio to help go to sleep." Health and Well-being Improvement "Health improved." "Improvements in sleep." "Goal is to lessen pain. But life events that increase spikes in cortisol levels have made the progress slow." "I have meditated a handful more times after the session than I was capable of beforehand." "Reduction in the sharpness of the pain. Hoping to get to a point where I don't notice it as much." Coping and control Self-Care and Coping Strategies "Coping with the pain a little bit better and not letting it slow me down, powering through the pain." "Having material to tackle stress to help my chronic pain." "Being more mindful. Trying to use meditation and bio feedback." "I have a plan now and don't feel so helpless when I'm having a bad day with my pain." "Because my back pain is acute, I was taking the class to help me should my pain come back and last for a longer period of time." Cognitive and Emotional Coping "Developing more positive self talk." "A more positive outlook." "I was given skills to use beyond listening to the tape." "When my pain starts to ramp up, sometimes I start breathing rapidly and my pulse gets high. I hyperventilate." "I don't understand this question. I anticipated having pain relief and I do have pain relief." Personal Empowerment and Control "Feeling like I have some control." "I have a plan now and don't feel so helpless when I'm having a bad day with my pain." "I hoped to learn how to have some control over chronic pain." "Feeling like I have some control over the situation has been empowering." "Being able to take control of my pain management without solely relying on medication." Separation from pain Pain Acceptance and Mental Separation "I think the improvements I've made were due to my being better able to distinguish 'my pain' from 'myself'." "Learning how to manage the pain when it reoccurs." "I hoped to learn how to have some control over chronic pain." "Feeling like I have some control." "I don't know if I anticipated much improvement at this point." Disengagement from Pain "Not focusing on pain and limited activity. Developing more positive self talk." "I think less about my pain." "I wanted a way to train my brain to think of something other than my chronic pain. The meditation is working well for that." "I thought it would help be more with taking my mind off the pain." "To learn relaxation technique that would take my mind away from the pain." Active skills and methods for managing pain Exercise and Physical Activity "Just to take my mind off knee replacement surgery pain and I have used the audio file many times since I participated in the class." "Reduce the pain in my knee." "Muscle spasms as a result of cervical spine surgery are less frequent." "To get motivated to exercise." "Less back pain." Anxiety and Stress Reduction "Improvement in

brain fog and fewer migraines." "I have been working to actively lower my response to feelings of stress." "To calm anxiety flares and incorporate relaxation techniques." "Being able to use deep breathing to help with anxiety and depression." "I have been able to reverse a few times of extreme anxiety that left me physically sick." Increased Awareness and Mindfulness "Being more mindful. Trying to use meditation and bio feedback." "Listening to the audio has helped me focus on my breathing in different parts of my body and helped me to relax." "Just a reminder to keep mindful of my pain and try to use my breathing exercises." "I find myself Stoppard taking deep relaxing breaths throughout the day." "I am more relaxed and can calm myself in certain situations." Challenges Adverse Feedback on Program Delivery "Having previously attended other and different online CBT help sessions, I've found that online doesn't work for me. I need in-person." "I still want to continue meditating along with the sound; however, I found that the voice-over in the audio clip you provided to be highly irritating." "I have not been able to implement these changes in my daily living." "This is an awkwardly worded question, so I find it hard to answer." "I feel the same as I did before the program." Challenges in Implementing Program Techniques "I have not been able to implement these changes in my daily living. I've been dealing with my parents, and driving them to Dr. appointments." "Having previously attended other and different online CBT help sessions, I've found that online doesn't work for me." "Anticipating surgery, I really didn't follow the program." "I really had no idea what to expect." "Nothing the pain will go away." Reflecting on Program Expectations vs. Reality "This is an awkwardly worded question, so I find it hard to answer. I tried not to anticipate too much because my experience has been that many of the things I've tried to reduce my pain have not worked as well as I hoped." "I don't know if I anticipated much improvement at this point." "I feel the same as I did before the program." "I really had no idea what to expect." "I don't know. I am always aware of the discomfort that I have due to PI-LL mismatch." Barriers and limitations Need for Program Adaptation and Personalization "Having previously attended other and different online CBT help sessions, I've found that online doesn't work for me." "I still want to continue meditating along with the sound; however, I found that the voice-over in the audio clip you provided to be highly irritating." "CBT only works with continued repetition, and one session isn't nearly enough." "There's tv advertising (Calm) and much info online about binaural 'beats.' I would find it helpful to know more about using the different types of sound." "I would have preferred sound only with no voice." Limitations Due to Life Circumstances "I have not been able to implement these changes in my daily living. I've been dealing with my parents and driving them to Dr. appointments." "Most days I'm traveling from early morning to early evening." "Because of my back pain, I was taking the class to help me should my pain come back and last for a longer period of time." "It's only been 5 weeks and she said people didn't see results until after 11 weeks." "I really had no idea what to expect." Next steps Seeking Information and Guidance "I would find it helpful to know more about using the different types of sound (brown, green, pink noise?) with some recommendations for deciding among all the choices now online." "Of course, I'd really like to download free selections." "To understand the biology and psychology of pain." "I was hoping to get enough relief to be able to live life." "I don't understand this question. I anticipated having pain relief and I do have pain relief." Desire for Non-Medication Approaches "Finding an alternative to

medicine for pain relief." "When my pain starts to ramp up, sometimes I start breathing rapidly and my pulse gets high. I hyperventilate. Now when my pain starts I immediately listen to the binaural audio file." "Being able to use deep breathing to help with anxiety and depression." "Breaking the stress causing pain, and pain causing more stress cycle." "Relaxation/calming techniques to help with constant feeling of fight or flight that results in chronic fatigue." Seeking Alternative Pain Relief Methods "Finding an alternative to medicine for pain relief." "Binaural sound for relaxation and meditation seems to offer the most promise for me." "I was hoping to relieve my pain; getting another shot next week to help with pain." "There's tv advertising (Calm) and much info online about binaural 'beats.'" "I had a medication change that was extremely effective."

ChatGPT said:

ChatGPT

Based on the detailed feedback from participants regarding the benefits of the pain management program, a second thematic analysis can be summarized as follows:

Second Thematic Analysis Summary: Benefits of the Educational Pain Management Program

The second thematic analysis of participant feedback highlights the substantial benefits of the educational pain management program in various aspects of health and well-being, specifically targeting pain management, relaxation, mindfulness, sleep quality, and overall health and wellness.

Improved Pain Management: Participants reported a noticeable reduction in both the intensity and frequency of pain. Chronic conditions such as headache pain, joint pain, and overall pain levels were mitigated, with some participants noting the ability to reframe their pain as a 'safe sensation,' leading to relief.

Increased Relaxation and Mindfulness: The program has been effective in enhancing relaxation and mindfulness among participants. Individuals have become more adept at relaxing despite pain, with techniques taught in the program helping to combat the chronic fatigue associated with a constant fight or flight response.

Enhanced Sleep Quality: Improvements in sleep were a significant benefit, with participants reporting better sleep quality and an easier time falling asleep, especially after sessions. The use of audio aids to facilitate sleep was frequently mentioned.

Health and Well-being Improvement: Overall health improvements were noted, including better sleep and reduced sharpness of pain. Participants expressed a desire to continue making progress in managing life events that can cause spikes in stress and, consequently, pain levels.

Coping and Control: The program provided participants with self-care and coping strategies, empowering them to better manage their pain and not allow it to hinder their daily activities. This also led to the development of more positive self-talk and a positive outlook on life, which are crucial for cognitive and emotional coping.

Separation from Pain: A theme of pain acceptance and mental separation emerged, with participants learning to distinguish 'my pain' from 'myself,' indicating a form of mental compartmentalization that aids in managing recurring pain.

Active Skills and Methods for Managing Pain: Physical activities and exercises were employed as distractions from pain related to conditions such as knee replacement surgery or back pain. Furthermore, a reduction in anxiety and stress, through techniques such as deep breathing, contributed to an overall decrease in migraines and 'brain fog.'
Challenges: Despite the benefits, participants also reported challenges with the program delivery, such as a preference for in-person sessions over online formats and some adverse feedback regarding the program's audio components. Implementing techniques in daily life was also a challenge, especially when life circumstances, such as caring for family members, became a barrier.
Barriers and Limitations: Some participants felt the need for the program to be more adaptable and personalized, and they struggled with the limitations imposed by their individual life circumstances. A need for continued repetition of cognitive-behavioral therapy (CBT) was also noted.
Next Steps: Participants expressed a desire for additional information and guidance on non-medication approaches to pain relief, including sound therapy with binaural beats and deep breathing techniques. There was also an interest in understanding the biology and psychology behind pain, indicating a proactive stance toward managing their conditions.

In conclusion, the educational pain management program has been instrumental in providing participants with a variety of tools and techniques to manage their pain more effectively. The program has fostered a sense of personal empowerment and control over pain, leading to improved physical and emotional well-being. While challenges and barriers remain, the feedback underscores the participants' desire for continued support and exploration of alternative pain relief methods that extend beyond medication.
2/2
4
You said:

	Use of Audio Tools for Pain Management	"The audio App, Calm was helpful."<br>"Most helpful was the idea of using binaural sound meditation."<br>"I also prefer the audio especially at night."<br>"The calm, steady voice of the narrator was effective."<br>"The Binaural Relaxation was my favorite takeaway."
Mindset	Empowerment and Control Over Pain	"I got a feeling of empowerment which I feel that I have been able to keep with me."<br>"Using my own abilities to control pain."<br>"That I can partially control my pain, especially when I am at rest."<br>"Offered additional tools I did not have and gave me a better understanding that I was not alone in dealing with pain."<br>"For me it came at a perfect time and has been very helpful."
	Positive Thinking and Focus Shift	"Focus on trying to enjoy things a little better and not focus on pain."<br>"Optimistic and hopeful message. Reminder to use positive self talk."<br>"Redirect thinking about pain and its consequences."<br>"Making me focus and think about things that help reduce my pain."<br>"I learned a lot - but the best has been my use of the meditation app."
Technique	Relaxation Techniques	"I think it was helpful to just learn the relaxation techniques."<br>"The relaxation exercises."<br>"The relaxation tape and positive thinking."<br>"The instructor was pleasant and helpful. The relaxation techniques will help in the future."<br>"Helps me to relax and lessen the pain when trying to get to sleep."
	Meditation and Mindfulness Techniques	"Considering meditation like medicine helped me immensely."<br>"I guess, the meditation, and breathing techniques."<br>"Mental outlook and the meditation."<br>"Understanding the research, and finding appropriate meditations to help calm my nervous system."<br>"The entire process is new to me. I had not tried anything to reduce the pain other than the occasional use of Tylenol."
	Breathing Techniques	"I thought the breathing exercises were of a big help to me."<br>"The power of my breath and how I can distract my brain while experiencing pain."<br>"Just taking time to breathe really helps me and focusing on it."<br>"I remember to use my breathing to relax during stressful times."<br>"Focusing on slowing my breath. Many years ago I realized that a key indicator of my pain ramping up was shallow breathing."
Community and space	Community and Shared Experience	"Being able to share what we're going through."<br>"I realized that there were a lot of other people who had the same issues."<br>"Knowing that others also feel the same way I do, and reframing the shame I feel when I can't do things all the time."<br>"It was helpful learning that pain not only affects me emotionally but physically. It helped me to be in a group to see that others are dealing with pain like me- it made me feel less isolated."<br>"The class motivated me to actively continue to search for other ways to help myself."
	Emotional Coping and Management	"Offered additional tools I did not have and gave me a better understanding that I was not alone in dealing with pain."<br>"Knowing that others also feel the same way I do, and reframing the shame I feel when I can't do things all the time."<br>"That your emotions worsen pain."<br>"The relaxation tape and positive thinking."

Use the same format to analysis this their thematic analysis: Use of Audio Tools for Pain Management "The audio App, Calm was helpful." "Most helpful was the idea of using binaural sound meditation." "I also prefer the audio especially at night." "The calm, steady voice of the narrator was effective." "The Binaural Relaxation was my favorite takeaway." Mindset Empowerment and Control Over Pain "I got a feeling of empowerment which I feel that I have been able to keep with me." "Using my own abilities to control pain." "That I can partially control my pain, especially when I am at rest." "Offered additional tools I did not have and gave me a better understanding that I was not alone in dealing with pain." "For me it came at a perfect time and has been very helpful." Positive Thinking and Focus Shift "Focus on trying to enjoy things a little better and not focus on pain." "Optimistic and hopeful message. Reminder to use positive self talk." "Redirect thinking about pain and its consequences." "Making me focus and think about things that help reduce my pain." "I learned a lot - but the best has been my use of the meditation app." Technique Relaxation Techniques "I think it was helpful to just learn the relaxation techniques." "The relaxation exercises." "The relaxation tape and positive thinking." "The instructor was pleasant and helpful. The relaxation techniques will help in the future." "Helps me to relax and lessen the pain when trying to get to sleep." Meditation and Mindfulness Techniques "Considering meditation like medicine helped me immensely." "I guess, the meditation, and breathing techniques." "Mental outlook and the meditation." "Understanding the research, and finding appropriate meditations to help calm my nervous system." "The entire process is new to me. I had not tried anything to reduce the pain other than the occasional use of Tylenol." Breathing Techniques "I thought the breathing exercises were of a big help to me." "The power of my breath and how I can distract my brain while experiencing pain." "Just taking time to breathe really helps me and focusing on it." "I remember to use my breathing to relax during stressful times." "Focusing on slowing my breath. Many years ago I realized that a key indicator of my pain ramping up was shallow breathing." Community and space Community and Shared Experience "Being able to share what we're going through." "I realized that there were a lot of other people who had the same issues." "Knowing that others also feel the same way I do, and reframing the shame I feel when I can't do things all the time." "It was helpful learning that pain not only affects me emotionally but physically. It helped me to be in a group to see that others are dealing with pain like me- it made me feel less isolated." "The class motivated me to actively continue to search for other ways to help myself." Emotional Coping and Management "Offered additional tools I did not have and gave me a better understanding that I was not alone in dealing with pain." "Knowing that others also feel the same way I do, and reframing the shame I feel when I can't do things all the time." "That your emotions worsen pain." "The relaxation tape and positive thinking." "I am under extreme stress at this point in my life, so spending time with the binaural recording and learning the other coping ideas have been good things to take away from the program." Knowledge Understanding Pain and Its Effects "Understanding the power of the brain. I never thought about it before this session." "You mean the class, right. It was informative and helpful understanding the effects chronic pain has on your brain and life." "The science of pain, and its effect on the brain." "The science behind the physical changes with anxiety and how similar they are to the pain response." "I learned that your brain can not process pain and other thoughts at the same time, so if you can get your

brain to focus on something else it will help with pain relief." Educational Impact and Knowledge Gain "Understanding the research, and finding appropriate meditations to help calm my nervous system." "It was informative and helpful understanding the effects chronic pain has on your brain and life." "The science of pain, and its effect on the brain." "The science behind the physical changes with anxiety and how similar they are to the pain response." "Learning how to rethink pain." Tools and approaches Coping Strategies and Practical Tools "The tips to help lower stress and cope with pain." "The worksheets helping us to lay out an action plan, reframing unhelpful pain thoughts, reframing to 'best friend talk,' and coming up with a list of self-soothing actions." "A concrete plan - something constructive to do." "Learning how to rethink pain." "The after class materials were very helpful to get through pain afterwards." Personal Growth and Self-Reflection "Making me focus and think about things that help reduce my pain." "I got a feeling of empowerment which I feel that I have been able to keep with me." "The worksheets helping us to lay out an action plan, reframing unhelpful pain thoughts, reframing to 'best friend talk,' and coming up with a list of self-soothing actions." "The segment dealing with the binaural audio file. Any additional info regarding this type of therapy would be helpful." "The study on the use of binaural beats. Also filling out the worksheet before the workshop caused me to acknowledge how much guilt I felt about not being able to be the partner I want to be to my husband, because of my pain and how it changed our lives." Technological Tools for Pain Management "The audio App, Calm was helpful." "Most helpful was the idea of using binaural sound meditation." "The Binaural Relaxation was my favorite takeaway." "The binaural meditation app that was provided post meeting has been most helpful to me. I use it daily." "The audio file is a great way to concentrate and be mindful about relaxing." Self-awareness Self-Assessment and Body Awareness "Importance of self-assessment and noting how my body responds to different activities." "I learned to recognize my pain and actually respond to it." "I learned to listen to my body and recognize symptoms of anxiety as pain." "Taking the time to refocus." "I got a feeling of empowerment which I feel that I have been able to keep with me." Adapting to Physical Limitations and Recovery "For me it came at a perfect time and has been very helpful. It is hard to find time to squeeze everything into your day, with PT 2 to 3 times a day, although when I do I feel so much better." "I had robot assisted surgery and the pain in a lot less than I expected, I would say it has never passed the 4 level and that it is in the 2-3 level." "Turns out I had Lyme disease and was treated which reduced a lot. I also had low cortisol - Secondary Adrenal Insufficiency and am now on medication which also reduced some pain." "The entire process is new to me. I had not tried anything to reduce the pain other than the occasional use of Tylenol." "I wish I could listen to the calming tones but I cannot tolerate them with my migraines. Maybe in the future I could try again."

ChatGPT said:

ChatGPT

The third thematic analysis of participant feedback on the educational pain management program uncovers further insights into the experiences and outcomes for individuals participating in the program, highlighting the impact on their pain management strategies, mental state, community involvement, knowledge acquisition, and use of tools and self-awareness techniques.

Third Thematic Analysis Summary: Educational Pain Management Program

Use of Audio Tools for Pain Management: Participants found audio tools, specifically the Calm app and binaural sound meditation, to be particularly helpful. The soothing quality of the audio narration and the effectiveness of binaural relaxation techniques stood out as key takeaways for managing pain, especially at night.

Mindset: Empowerment and Control Over Pain: The program instilled a sense of empowerment in participants, providing them with the belief and ability to exert control over their pain, especially during restful periods. It offered new tools and a reassurance that they were not alone in their struggle with pain, which came at a critical time for many.

Positive Thinking and Focus Shift: A shift in focus towards positive thinking was another significant benefit. Participants reported being encouraged to enjoy life more and redirect their thoughts away from the pain and its consequences. The optimistic messaging and the use of positive self-talk were effective in altering their focus and reducing pain.

Technique: Relaxation Techniques: Learning relaxation techniques was beneficial for participants, helping them to manage stress and pain more effectively. The relaxation exercises were particularly useful when trying to sleep, indicating their immediate applicability.

Meditation and Mindfulness Techniques: Considering meditation as a form of medicine was a breakthrough for some participants. The meditation and breathing techniques provided mental relief and were key in helping to manage pain.

Breathing Techniques: Participants highlighted the significance of breathing exercises. The awareness of the power of breath and its ability to distract from pain was noted as a considerable help.

Community and Space: Community and Shared Experience: The shared experiences within the program's community allowed participants to feel less isolated and motivated them to continue seeking pain relief methods. Sharing struggles with others provided emotional relief and a sense of belonging.

Emotional Coping and Management: The program equipped participants with tools for emotional coping and helped them reframe negative emotions associated with pain. Learning about the interplay between emotions and physical pain was valuable for their overall coping strategy.

Knowledge: Understanding Pain and Its Effects: The scientific information about pain and its effects on the brain provided by the program was eye-opening for participants. It offered a new understanding of the power of the brain in pain management.

Educational Impact and Knowledge Gain: Gaining knowledge about the research behind pain management strategies empowered participants to find suitable meditations and rethink their approach to pain, equipping them with a better understanding of chronic pain's impact on life.

Tools and Approaches: Coping Strategies and Practical Tools: Participants appreciated the practical tools and strategies provided by the program, such as worksheets for action plans and reframing pain thoughts. These tools helped them develop a constructive approach to dealing with pain.

Personal Growth and Self-Reflection: The program fostered personal growth and self-reflection, encouraging participants to focus on reducing pain and empowering them to maintain a positive outlook.
Technological Tools for Pain Management: The provision of technological tools, particularly the binaural meditation app, was mentioned as helpful. Participants found these tools valuable for daily mindfulness and relaxation practice.
Self-awareness: Self-Assessment and Body Awareness: The program heightened participants' self-assessment and body awareness, enabling them to better understand their pain responses and to adopt proactive measures in response to pain.
Adapting to Physical Limitations and Recovery: Participants reported the program's timing was optimal, aiding in their recovery and adaptation to physical limitations. For some, the program coincided with medical treatments for underlying conditions, which together contributed to pain reduction.

In conclusion, this third thematic analysis underscores the program's effectiveness in enhancing pain management through the use of audio tools, fostering a positive and empowered mindset, teaching practical relaxation and meditation techniques, building a supportive community, expanding pain-related knowledge, offering practical coping tools, and promoting self-awareness and personal growth. Despite some challenges with implementing certain techniques, the overall sentiment reflects a beneficial impact on the participants' ability to manage pain and improve their quality of life.
